# The abundance of homoeologue transcripts is disrupted by hybridization and is partially restored by genome doubling in synthetic hexaploid wheat

**DOI:** 10.1186/s12864-017-3558-0

**Published:** 2017-02-10

**Authors:** Ming Hao, Aili Li, Tongwei Shi, Jiangtao Luo, Lianquan Zhang, Xuechuan Zhang, Shunzong Ning, Zhongwei Yuan, Deying Zeng, Xingchen Kong, Xiaolong Li, Hongkun Zheng, Xiujin Lan, Huaigang Zhang, Youliang Zheng, Long Mao, Dengcai Liu

**Affiliations:** 1 Triticeae Research Institute, Sichuan Agricultural University, Chengdu, Sichuan 611130 China; 2 National Key Facility for Crop Gene Resources and Genetic Improvement, Institute of Crop Science, Chinese Academy of Agricultural Sciences, Beijing, 100081 China; 3 Biomarker Technologies Corporation, Beijing, 101300 China; 4 Key Laboratory of Adaptation and Evolution of Plateau Biota, Northwest Institute of Plateau Biology, Chinese Academy of Sciences, Xining, 810001 China

**Keywords:** Genome duplication, Interspecific hybridization, Polyploidy, Synthetic wheat, Transcriptome evolution

## Abstract

**Background:**

The formation of an allopolyploid is a two step process, comprising an initial wide hybridization event, which is later followed by a whole genome doubling. Both processes can affect the transcription of homoeologues. Here, RNA-Seq was used to obtain the genome-wide leaf transcriptome of two independent *Triticum turgidum* × *Aegilops tauschii* allotriploids (F1), along with their spontaneous allohexaploids (S1) and their parental lines. The resulting sequence data were then used to characterize variation in homoeologue transcript abundance.

**Results:**

The hybridization event strongly down-regulated D-subgenome homoeologues, but this effect was in many cases reversed by whole genome doubling. The suppression of D-subgenome homoeologue transcription resulted in a marked frequency of parental transcription level dominance, especially with respect to genes encoding proteins involved in photosynthesis. Singletons (genes where no homoeologues were present) were frequently transcribed at both the allotriploid and allohexaploid plants.

**Conclusions:**

The implication is that whole genome doubling helps to overcome the phenotypic weakness of the allotriploid, restoring a more favourable gene dosage in genes experiencing transcription level dominance in hexaploid wheat.

**Electronic supplementary material:**

The online version of this article (doi:10.1186/s12864-017-3558-0) contains supplementary material, which is available to authorized users.

## Background

Allopolyploidization is an important driver of plant speciation [1]. In the initial hybridization two (or more) distinct genomes are combined within a single nucleus, with fertility subsequently being restored by a whole genome doubling (WGD) [2, 3]. Although a *de novo* polyploid carries a complete copy of each of its constituent genomes, the early post-allopolyploidization generations typically experience a spectrum of genomic changes [4–8], a response to “genome shock” [9]. By definition, allopolyploids carry more than one copy of any given single copy gene, but these copies are not necessarily transcribed and expressed in an additive fashion [10–12]. The genomic perturbations and alterations in individual homoeologue transcription induced by the allopolyploidization process generate a level of genetic novelty which gives opportunities for selection [11, 13, 14].

The independence of the hybridization and WGD events has allowed for an experimental demonstration that it is the former which induces most of the transcriptomic changes associated with alloployploidization, possibly as a result of its relaxation of transcriptional regulation [15–19]. However, in *Senecio* sp., WGD was also found have an obvious effect, since the extent of the transcriptional changes appeared to be less marked in the allohexaploid setting than in its allohaploid progenitor [20, 21]. In a small number of cases, the opposite has been demonstrated, i.e., that allopolyploidization can further disrupt gene transcription [22].

The most recent allopolyploidization event in the evolution of bread wheat (*Triticum aestivum*) involved the formation of a hybrid between a cultivated form of the AB genome allotetraploid species *T. turgidum* and the D genome diploid goatgrass *Aegilops tauschii* [23–25]. Comparisons of transcription profiles between re-synthesized hexaploid wheat and its progenitors have suggested that additivity between homoeologues is commoner than non-additivity [26, 27]. However, the phenomenon of parental expression level dominance (ELD) has also been documented: this relates to the situation where, for a set of homoeologues transcribed at different levels in the parents, the total expression level of these homoeologues in the progeny is statistically similar to that of one parent [28]. The suggestion has been made that in wheat, ELD may underlie some of the vigour and adaptability of the species [27, 29]. The acquisition of draft genome sequences for bread wheat and its A and D genome donors (respectively, *T. urartu* and *A. tauschii*) provides an unparalleled opportunity to track homoeologue transcript abundances during allohexaploidization [30–32]. Here, the global gene transcription profiles in the leaf of allotriploid hybrids and their WGD-derived allohexaploid product have been compared. The picture which emerges is one where both the hybridization and WGD events have an influence over the transcriptome.

## Results

### The phenotype of de novo synthesized hexaploid wheat, its allotriploid form and its progenitors

**Fig. 1:**
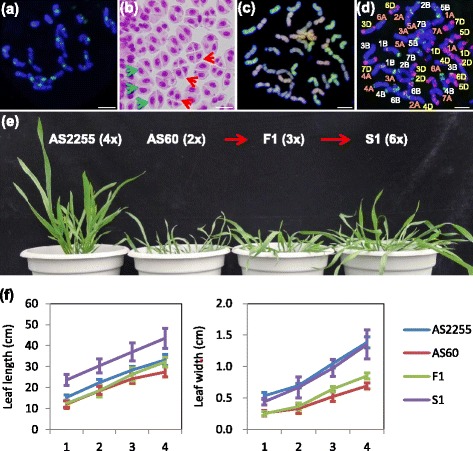
The morphology and cytology of *T. turgidum* AS2255 (AABB), *A. tauschii* AS60 (DD), the allotriploid AS2255 × AS60 (ABD) and the derived allohexaploid (AABBDD). **a** Fluorescent in situ hybridization (FISH) analysis of the 21 univalents presents at meiosis metaphase I in the meiocyte of an allotriploid plant. The probe 6C6-3 hybridizing to the centromeres fluoresced green. Bar: 10 μm. **b** Allotriploid pollen mother cells comprise a mixture of dyads (*green arrowheads*) and tetrads (*red arrowheads*). **c** Multi-colour genomic in situ hybridization of a root tip mitotic cell from an allohexaploid plant, showing 2*n =* 6*x* = 42. **d** Sequential multi-colour FISH of a root tip mitotic cell from an allohexaploid plant, showing that chromosomes of the A, B and D genome were all represented on basis of probes pSc119.2 (*green*), pAs1 (*red*), and pTa71 (*yellow*). **e** Morphology of 120 day old plants of AS2255, AS60 and their derived allotriploid (F1) and allohexaploid (S1). **f** Leaf width and length of the first four leaves of the plants. Whiskers indicate SD (allotriploid: *n =* 7, AS2255, AS60 and allohexaploid: *n =* 12)

The F1 plants (allotriploid; 3*x* = 21, ABD) were generated from a wide cross between either *T. turgidum* (2*n* = *4x* = 28, AABB) ssp. *turgidum* accession AS2255 or ssp. *durum* LDN (cv. Langdon) and *A. tauschii* accession AS60 (2*n* = 2*x* = 14, DD). The chromosomes in the allotriploid metaphase I meiocytes typically formed 21 univalents (Fig. 1a and Additional file 1: Figure S1a), but a significant number of immature microspores formed a dyad, diagnostic of the presence of an unreduced gamete (Fig. 1b and Additional file 1: Figure S1b). Zygotes derived from the union of two unreduced gametes represent a WGD event, producing an allohexaploid (2*n* = 6*x* = 42, AABBDD) embryo (Fig. 1c, d and Additional file 1: Figure S1c, d).

The allotriploid plants were less vigorous than their *T. turgidum* progenitor (Fig. 1e and Additional file 1: Figure S1e). Their leaf length and width were intermediate between the two parents’ during the early stage of the plants’ development (Fig. 1f). In contrast the leaf length of the allohexaploid plants in the first generation (S1) was greater than their *T. turgidum* progenitor’s in both combinations (Fig. 1f and Additional file 1: Figure S1f; Student’s *T*-test, *P* < 0.01). As allotriploid and allohexaploid S1 plants were most distinguishable at this stage (Fig. 1e) and there were no noticeable differences in morphology among S1-S4 euploid plants (Additional file 1: Figure S1e), we collected leaves at this stage from S1 plants to isolate RNA for sequencing. For all S1 plants used, root tip cells were checked to make sure they contained a complete set of chromosomes without structure variation.

### Homoeologue discrimination and transcript abundance in the allotriploid, allohexaploid and parental plants

RNA-Seq analysis was applied to RNA extracted from allotriploid, allohexaploid (S1) and the progenitor plants (Additional file 2: Dataset S1). The number of clean reads ranged from 46.7 to 89.4 million, and a mean of 77.3% of these were mappable to the cv. Chinese Spring (CS) draft genome sequence. After a stringent filtering step using the method by Pfeifer et al. [33], an average of 36% of the mapped reads could be allocated to one of the homoeologues. The transcript abundance of each homoeologue was then quantified using HTSeq-count [34], based on the high confidence gene (HC1-HC4) dataset [32], comprising 99,386 gene models. Counts were expressed as fragments per kilobase of exon model per million mapped base pairs (FPKM) [35]. Only homoeologues/genes showing an FPKM greater than unity in at least one sample within a lineage were retained. Abundances of biological duplicates proved to be strongly correlated (R^2^ = 0.87–0.96 in the AS2255 × AS60 combination, in Additional file 1: Figure S2a). A correlation dendrogram showed that the allotriploid was more closely related to the allohexaploid plant than to its tetraploid progenitor, with the diploid progenitors appearing as outliers (Additional file 1: Figure S2b, c), as would be expected from the various plants’ genome constitutions.

Although the transcripts of the two *T. turgidum* accessions mapped for the most part to the A and B genome chromosomes, a small number were associated with a D genome location; similarly, a few of the *A. tauschii* transcripts apparently mapped to an A or B genome chromosome location (Additional file 1: Figure S2d, e). These locations are likely artefacts, deriving from polymorphism between the parental genome sequences and corresponding subgenome sequences of CS. These reads were discarded. The median relative abundance of the ~20,000 genes for which a read of each homoeologue was obtained was close to unity in both hybridization lineages, while the peak value for FPKM was >100 (Additional file 1: Figure S2f). Applying a threshold of FPKM >1, 15,418 (AS60) and 37,321 (LDN × AS60 allohexaploid) genes were identified for each species (Additional file 1: Table S1). Based on the criteria 90% sequence identity and 90% alignment, the CS gene models could be classified as 8,339 triplets (each of the three homoeologues represented), 8,338 duplets (two of the three homoeologues represented) and 47,622 singletons (only one homoeologue represented). An average of 60% triplets, 50% duplets and 30% singletons were identified in the transcriptomes of hybridization combination (Additional file 1: Table S2). For the most part the two hybrid lineages behaved similarly. What follows relates mostly to the AS2255 × AS60 combination, unless indicated otherwise. Although the identity of the *T. turgidum* parent had little influence over the pattern of homoeologue transcription, there were some differences in the estimated overall gene number. Data relating to the LDN × AS60 combination are given in Additional file 3: Dataset S2, Additional file 4: Dataset S3, Additional file 5: Dataset S4 and Additional file 1: Figures S3–S5 .

### WGD restored homoeologue transcript abundances disturbed by hybridization

**Fig. 2:**
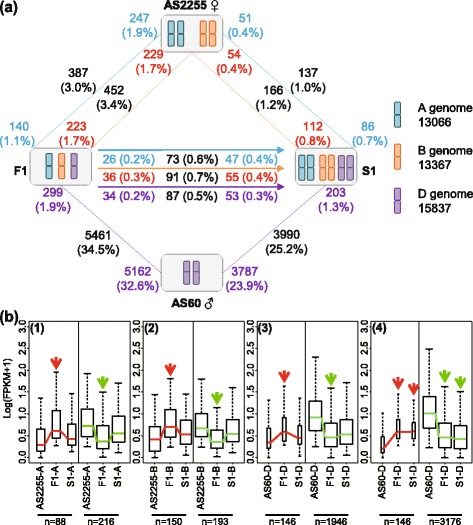
Variation in the transcription of homoelogues as a result of allotriploidization and WGD in the AS2255 × AS60 lineage. **a** Differentially transcribed homoeologues. The number next to the symbol for the species represents the number of differentially up-regulated homoeologues vs. the neighboring species linked by a line. A consistent colour has been used to refer to each genome (A genome: *blue*, B genome: *yellow*, D genome: *purple*). Numbers in the middle of each line represent the total numbers of differentially transcribed homoeologues (*black*). **b** Boxplots illustrating the effect of allotriploidization and WGD on transcript abundance: homoeologues from (1) the A genome, (2) the B genome, (3) the D genome. Differentially transcribed D genome homoeologues between the allotriploid and parent that were transmitted into allohexaploid are used as controls (4). Boxes span the data range between the first and third quartiles, and the median is represented as a horizontal line. Whiskers extend to the most extreme data point, which is no more than 1.5 times the interquartile range away from the first and third quartiles. The widths of the boxes are proportional to the gene numbers

In all, 32.6% of the set of D-subgenome homoeologues (5,162 out of 15,837) in the allotriploid plants compared to diploid parents AS60 of AS2255 × AS60 combination were down-regulated. The equivalent frequency for the A and B genome homoeologues compared to tetraploid parents AS2255 was, respectively, only 1.9% and 1.7% (Fig. 2a; Additional file 6: Dataset S5). The frequency of up-regulated homoeologues was small for all three genomes (1.1% for A, 1.7% for B and 1.9% for D). An even smaller proportion (0.7% for each genome) was altered in the move from ABD to AABBDD (Fig. 2a; Additional file 7: Dataset S6). However, 3,787 D-subgenome homoeologues appeared to be down-regulated when the contrast was made between the allohexaploid and AS60, a number which was ~25% less than in the contrast between the allotriploid and AS60. The proportion of A and B genome homoeologues down-regulated in the allohexaploid was also lower (0.4% *vs* ~1.8% in the allotriploid *vs* AS60 contrast). The implication is that WGD effectively reduced the number of differentially transcribed homoeologues, probably by minor changes but non-significant difference on statistical analyzing using FDR-adjusted *p*-value below 0.05 that potentially cause inflated comparisons of expression level differences. In order to confirm this suggestion we analyzed theses differential expressed genes only appeared between F1 and parents by boxplot analysis using R packages. This corrective effect was supported by the contrasting effects of hybridization and WGD on homoeologue transcript abundances: for example, 1,946 (38%) D-subgenome homoeologues were significantly down-regulated in the allotriploid plants (Fig. 2b, panel 3, right side), and their transcript abundances were marginally increased in allohexaploid (S1-D), as demonstrated by the upward shift in the median abundance. A similar contrasting effect was observed for homoeologues up-regulated in the allotriploid plants, even for the A and B genome homoeologues. At the same time, 3,176 D genome homoeologues remained down-regulated in the allohexaploid (Fig. 2b, panel 4). The large number of genes for which the level of transcription was corrected in this manner suggests that this is an important feature of the polyploid wheat transcriptome.

A further effect of WGD on the transcription of the set of unequally transcribed homoeologues (UTHs) was noted: this set represented 25% (1,358/5,460) of the genes defined in the *T. turgidum* parent AS2255 for which at least one of the homoeologues showed an FPKM greater than unity (Table 1); however this proportion rose to 38% in the allotriploid plants, falling to 22% in the allohexaploids. The decrease effect on UTH proportion from allotriploids to allohexaploids applied similarly to A genome vs D genome and B genome vs D genome genes in both lineages (Table 1).

**Table 1:** Changes in the transcript abundance of homoeologues induced by allotriploidization and WGD^*^

Genotypes		No. of homoeologues				No. of homoeologues				No. of homoeologues		
Parents AS2255 and AS60	Types	A > B	A < B	Total	Types	A > D	A < D	Total	Types	B > D	B < D	Total
		707 12.9%	651 11.9%	5460		825 13.8%	964 16.1%	5986		845 14.1%	1036 17.3%	5988
Allotriploid	A > B	621	0	1053 19.3%	A > D	554	4	971 16.2%	B > D	571	2	1020 17.0%
	A < B	0	569	1021 18.9%	A < D	1	561	941 15.7%	B < D	5	616	1014 16.9%
Allohexaploid	A > B	507	0	639 11.7%	A > D	417	1	571 9.5%	B > D	405	1	567 9.5%
	A < B	1	467	566 10.4%	A < D	0	415	537 9.0%	B < D	2	484	620 10.4%
Parents LDN and AS60	Types	A > B	A < B	Total	Types	A > D	A < D	Total	Types	B > D	B < D	Total
		518 9.4%	450 8.1%	5533		742 12.3%	602 10.0%	6037		783 13.0%	632 10.5%	6035
Allotriploid	A > B	355	0	718 13.0%	A > D	353	1	595 9.9%	B > D	362	1	620 10.3%
	A < B	0	309	657 11.9%	A < D	1	325	592 9.8%	B < D	2	357	692 11.5%
Allohexaploid	A > B	321	0	358 6.5%	A > D	257	0	292 4.8%	B > D	245	0	283 4.7%
	A < B	0	273	311 5.6%	A < D	0	220	294 4.9%	B < D	0	249	344 5.7%

^*^Values differ significantly (*P* < 0.05) following the application of the Benjamini-Hochberg multiple test correction. Based on the CS gene models, the triplet genes were used in the comparison of homoeologues between A-, B, and D-subgenomes. Only those that at least one of three homoeologues were expressed with FPKM >1 in parents (number of total) were analyzed

### WGD corrected the non-additive down-regulation of genes induced by hybridization

**Fig. 3:**
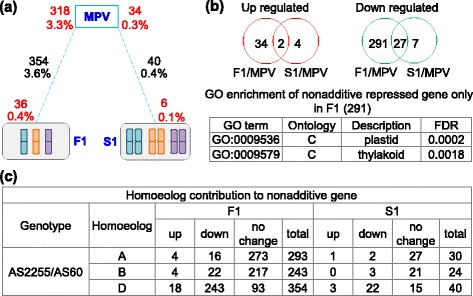
Non-additive transcription of genes in the allotriploid and allohexaploid in the lineage AS2255 × AS60. **a** Numbers of non-additively transcribed genes in the progeny compared to mid-parent value (MPV). The red numbers shown refer to genes up-regulated (*bottom*) or down-regulated (*top*) in the allotriploid (F1) and allohexaploid (S1). **b** The number of non-additive genes common to the allotriploid and allohexaploid. GO enrichment terms for the genes non-additively down-regulated in the allotriploid are shown below the figure. **c** Homoeologue expression patterns of non-additively expressed genes. “Up” and “down” refer to homoeologues differentially transcribed between the progeny and the parents, whereas “no change” implies that the transcription levels were statistically unchanged by either the allotriploidization or the WGD

Based on the CS gene models, it was possible to determine the transcript abundance of each gene in the parental lines and their allotriploid and allohexaploid derivatives (Additional file 8: Dataset S7). When mid-parent values were compared with those recorded in the allotriploid and allohexaploid plants, non-additivity was assignable to 354 genes (3.6%) of the genes in the allotriploids, the abundance of most of which (318/354) was below the mid-parent value (Fig. 3a). GO analysis showed that genes related to cellular component terms “plastid” and “thylakoid” were well represented (Fig. 3b). The D-subgenome homoeologues were especially affected: 243 (68.6%) were less abundant in the allotriploids than in AS60 (Fig. 3c). However, the transcript abundance of only 40 of these genes remained below the mid-parent value in the allohexaploid plants.

### ELD was frequent in both the allotriploid and allohexaploid plants

**Fig. 4:**
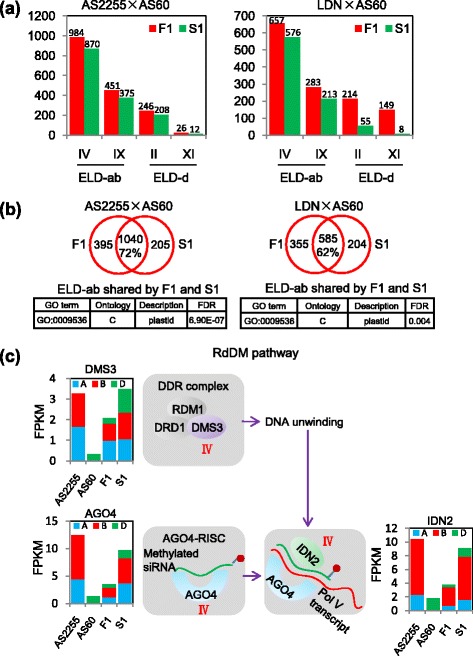
Parental expression level dominance (ELD) genes in the allotriploid and allohexaploid. **a** The number of genes with a transcription level similar to that in *T. turgidum* (ELD-ab genes) or that in AS60 (ELD-d genes) in both the AS2255 × AS60 and LDN × AS60 lineages. **b** The number of ELD-ab genes common to the allotriploid and allohexaploidand the associated enriched GO terms. **c** Genes encoding major components of the RNA-dependent DNA methylation pathway (DMS3, AGO4, and IDN2) were classified as ELD-ab genes. The histograms show the FPKMs of the relevant homoeologues in AS2255 (A genome *blue*, B genome *red*), AS60 (*green*), allotriploid (ABD) and allohexaploid (AABBDD)

There was a five fold greater number of ELD-ab genes (those showing a similar level of transcription in AS2255 and the allotriploid) than ELD-d genes (showing a similar level of transcription in AS60 and the allotriploid) (1,435 vs. 272, see Additional file 1: Table S3). In each case, the transcription level in the allotriploid was more similar to that of the parent showing the higher transcription level (ELD-ab: 984/1,435, ELD-d: 246/272; Fig. 4a). The pattern was largely retained at the allohexaploid: more than 72% of the ELD-ab genes behaved equivalently in the allotriploid and allohexaploid. Of the set of 1,245 ELD-ab genes identified in the allohexaploid, only 205 were not classed as ELD in the allotriploid (Fig. 4b). A GO analysis of the ELD genes shared by F1 and S1 revealed an enrichment for genes assigned to the cellular component “plastid”. Genes homologous to components of the RNA-dependent DNA methylation (RdDM) pathway, in particular those encoding ARGONAUTE 4 (*AGO4*), DEFECTIVE IN MERISTEM SILENCING 3 (*DMS3*), and the RNA-binding protein INVOLVED IN DE NOVO2 (*IDN2*), were among the ELD-ab genes identified in both the allotriploid and allohexaploid (Fig. 4c). Meanwhile, almost all (426/451) of ELD-ab genes identified in the allotriploid plant achieved this status thanks to the down-regulation of their D genome homoeologue (Additional file 1: Table S3).

### Complementation of genome-specific singletons in allotriploid and allohexaploid

**Fig. 5:**
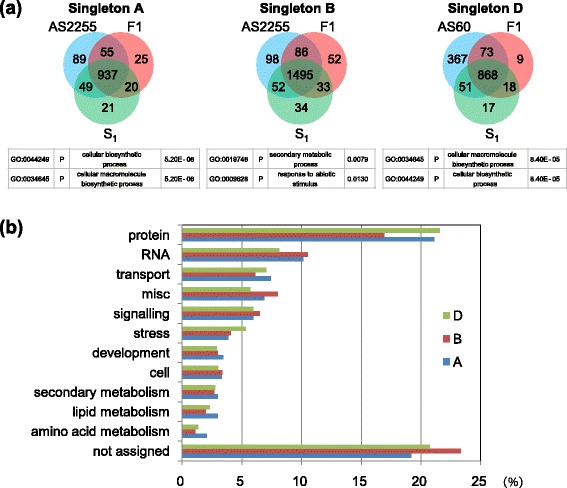
The transcription of singletons in the AS2255 × AS60 lineage. **a** Singletons classified according to genome origin; enriched GO terms found in the shared singleton genes are shown below the Venn diagram. **b** The function of singletons derived from the MapMan program

As referred to earlier, 53.3% of the 89,315 annotated CS genes were classified as singletons (Additional file 1: Table S2). Of the 13,505 singletons assigned to high confidence group 1 (the HC1 group listed in Additional file 9: Dataset S8) [32], over a quarter were associated with an FPKM greater than unity, and most were consistently transcribed in the AS2255 × AS60 allotriploid and allohexaploid (Fig. 5a). Among the 968 D-subgenome singletons transcribed in the allotriploid, 886 were also transcribed in the allohexaploid. A GO analysis revealed some enrichment in the processes “cellular macromolecule biosynthesis” and “cellular biosynthesis” (Fig. 5a). Some additional processes were enriched for the B genome singletons, including “secondary metabolism” and “response to abiotic stimulus”; and similarly for the A genome singletons with respect to “cellular biosynthesis”. A number of functions were represented among the set of singletons (Fig. 5b). The singletons identified in the allotriploid and allohexaploid of the other lineage (LDN × AS60) covered a similar set of functions (Additional file 1: Figure S5). Members of the set of D-subgenome singletons were more frequently down-regulated in both the allotriploid and allohexaploid (Additional file 1: Table S4).

## Discussion

Nascent allohexaploid wheat has been used to introduce the genetic divergence from the tetraploid and diploid progenitors into bread wheat. The poor vigour displayed by the allotriploid is greatly enhanced by WGD, restoring much of the phenotype exhibited by its tetraploid AABB progenitor. As a result, it has proved highly practical to exploit *de novo* synthetic hexaploid wheat as a bridge to enable the transfer of genetic material into the bread wheat genepool from its wild donors [36]. Understanding how homoeologues respond at the transcriptional level to both the allotriploidization event and then the subsequent WGD is of relevance if the maximum benefit is to be gained from synthetic hexaploids. The capacity to capture the entire transcriptome allows a global picture of gene reprogamming to be acquired, in contrast to earlier studies which relied on relatively low numbers of expressed sequences. Up to now, the focus of experiments aimed at characterizing homoeologue transcript abundances in polyploid wheat has been on established amphiploids [26, 29], an approach which ignores the possibility that alloploidization and WGD may have different, even opposing, effects. If genomic shock is a reality, it will be felt most intensely in the initial hybrid rather than in its subsequent derivatives, as was demonstrated here, where the extent of transcriptome reprogramming induced by the allotriploidization process was much greater than that induced by the subsequent WGD.

### The re-programming of D genome homoeologue transcription is a prominent characteristic of the allotriploid transcriptome

Hybridization appears to relax the regulation of transcription, thereby inducing differences between a hybrid and its progenitors with respect to the level of transcription of many genes. In diploids such as *Arabidopsis thaliana* [37] and rice [22], the sequence-based tagging of alleles is straightforward, but in polyploids such as *Brassica napus* [6], cotton [28] and wheat [29], the situation is complicated by the presence of homoeologues. Bread wheat has evolved from two independent and temporally well separated polyploidization events. The first of these, which occurred ca. 0.5 M years ago, involved the hybridization of *T. urartu* with an *Aegilops* species belonging to the *Sitopsis* section to form *T. turgidum*; the second was between a cultivated form of *T. turgidum* and *A. tauschii* and dates to some 0.01 M years ago [25]. Both events are likely to have a profound effect on the transcriptome. The asymmetry involved in combining a tetraploid with a diploid may account for the observation that it was the D genome homoeologues which suffered the greatest extent of down-regulation in the *de novo* allotriploid. One possibility is that the silencing involved is activated by the small RNA-mediated DNA methylation system, given that there appears to be a concentration of 24 nt RNAs around a number of genic loci in the D genome [27, 29]. Here, the data imply that D genome homoeologue regulation was the major contributor to the altered transcription patterns exhibited by the allotriploid plants. A major proportion of the non-additive transcribed genes and the ELD-ab genes were also associated with the D-subgenome homoeologues. Curiously, non-additive transcription in the allotriploid plants was for the most part reversed in the allohexaploids, whereas a large proportion of ELD genes maintained their status following the WGD. It is conceivable therefore that the former genes are in some way responsible for the inferior growth of the allotriploid plants, while the latter help to restore vigour to the allohexaploids.

### The restoration by WGD of parental homoeologue transcript abundance

WGD is required to restore fertility via the provision of pairs of homologous chromosomes. It also has the effect, as shown here, of reversing some of the disturbance to transcription induced by the hybridization; a similar effect has been noted in *Senecio × baxteri* when hybrids are created between tetraploid and diploid forms [20, 21]. In wheat, WGD appears to have a relatively small, non-genome specific effect on homoeologue transcription. It reduced the number of UTHs present, an effect which may contribute to the observed reduction in non-additively transcribed genes and hence to the restoration of hybrid vigour. This contrasts to the role of WGD in polyploid rice, in which WGD has been shown to disrupt transcription [22]. The high rate of maintenance of ELD status in the transition from allotriploid to allohexaploid (especially for the ELD-ab genes) may go some way to explaining the morphological similarity between the allohexaploid and tetraploid plants. Several of the ELD-ab genes were homologues of genes encoding RNA-dependent DNA methylation (*DMS3, AGO4*, and *IDN2*), which could imply that the maternal parent’s epigenetic modification system was maintained during the process of allohexaploidization.

### Singletons may represent a source of novel functionality

The bioinformatics-based analysis of the CS transcriptome suggested that over 50% of the gene content comprised singletons. This number may well represent an overestimate, since the current wheat gene model set was derived from an assembly of rather short reads [32]. A large number of the supposed singletons were found in the transcriptomes of both the allotriploid and allohexaploid, so they may well have a prominent effect on the phenotype of both hybrid forms. The combination of singletons from the three genomes may provide extra functions in hexaploid wheat. In maize, genes exhibiting so called “single parent transcription” - that is those which are silent in one of the parents of a hybrid, but transcribed both in the other parent and in the hybrid - have been shown to contribute materially to heterosis [38, 39]. In bread wheat, a number of genes underlying agronomic traits (notably disease resistances) have no known homoeologues [40], which confirms the notion that singletons in allopolyploids can be very important determinants of biological function.

## Conclusions

While WGD is believed to fix heterosis [41], here many of the non-additively transcribed genes identified in allotriploid wheat did not behave in this fashion at the allohexaploid level; a plausible interpretation of this phenomenon is that the effect of WGD can be hybrid specific. Rather, the persistence of ELD implies a role for this class of gene in determining the phenotype of hexaploid wheat. The combination of some ELD genes lends support to the “dominance model”, which proposes that the superior performance of a hybrid is due to the complementation of deleterious recessive alleles by dominant alleles at multiple genes [42].

## Methods

### Plant materials

Two independent *T. turgidum* (AABB, 2*n* = 4*x* = 28) accessions were used as the female parent in the wide cross: these were ssp. *durum* cv*.* Langdon (LDN) and ssp. *turgidum* accession AS2255. The male parent in each case was *A. tauschii* accession AS60 (DD, 2*n* = 2*x* = 14). Both the LDN × AS60 and AS2255 × AS60 allotriploid were produced, along with spontaneously doubled allohexaploid individuals. All three parents were shown to be highly homozygous on the basis of genotyping at 160 microsatellite loci [43]. The crosses required neither embryo rescue nor any hormone treatment. A total of, respectively, 4,035 and 810 allohexaploid grains from 8748 florets and 4802 florets were obtained following the generation of unreduced gametes from 11 LDN × AS60 and six AS2255 × AS60 allotriploid plants. They were grown as the first generation (S1) of allohexaploid plants. The S1 plants were self-pollinated to produce S2, S3, and S4 generations.

RNA was extracted from pot-grown plants raised outside under the local conditions used to grow winter wheat. Before potting, root-tips of individual allohexaploid plants were checked for euploidy by sequential multi-color genomic in situ hybridization (GISH) and fluorescence in situ hybridization (FISH) analyses, as described previously [3]. Out of the analyzed ~100 S2-S4 seeds in each combination, about 10% in LDN × AS60 and 20% in AS2255 × AS60 were aneuploid. No chromosome structural variation was observed except a chromosome fragment in a seed from AS2255 × AS60. Only euploid plants with a complete set of wheat chromosomes were selected for further analysis. The authenticity of triploid F1 hybrids was confirmed by meiotic observation of pollen-mother-cells, as described previously [3]. Measurements were taken of the length (from the base to the tip of a leaf) and width (at the widest part of the leaf) of the first to fourth leaves of seven AS2255 × AS60 allotriploid plants and 12 plants from each of the other lines.

### RNA sample preparation and transcriptome sequencing

The youngest fully expanded leaf at development stage 5 [44] was collected from three-five individuals of the parental, allotriploid and allohexaploid plants, snap-frozen in liquid nitrogen, and stored at −80 °C. Total RNA was extracted from the leaf tissue using an RNAprep Pure Plant kit (TIANGEN, Beijing, China), according to the manufacturer’s protocol. The integrity of the extracted RNA was validated using a 2100 Bioanalyzer (Agilent Technologies, Palo Alto, CA, USA). The RNA was used to construct and sequence 13 RNA-Seq libraries: two each from the AS2255 × AS60 and LDN × AS60 allotriploids, the AS2255 × AS60 and LDN × AS60 allohexaploids, AS2255 and AS60, and one from LDN. Paired end sequencing libraries (average insert size: 200 bp) prepared using a NEBNext® Ultra™ RNA Library Prep kit (New England Biolabs) and sequenced using a HiSeq2000 device (Illumina, San Diego, CA, USA) according to the manufacturer’s standard protocols. Raw RNA reads were de-multiplexed using bcl2fastq v1.8.4 (support.illumina.com/downloads/bcl2fastq_conversion_software_184.html). All contaminants and low quality reads were removed by enforcing a Q30 threshold of 80% and a maximum of 0.2% ambiguous base calls.

### Mapping of RNA-Seq reads to the reference genomes

TopHat v2.0.9 software (http://ccb.jhu.edu/software/tophat/index.shtml) was used to align the set of filtered reads from the two AS60 samples against the draft genome sequences of both *A. tauschii* (ftp://climb.genomics.cn/pub/10.5524/100001_101000/100054/D/Assembly/) and bread wheat (urgi.versailles.inra.fr/download/iwgsc/), allowing a maximum of two mismatches per alignment (parameters: −-bowtie1 -N 2 -r 40 --library-type fr-unstranded). An average of 77.6% of the reads was mappable to the former genome sequence (Additional file 1: Table S5), and of 74.2% to the latter one, indicating a similar mapping efficiency. As genic sequence proved to be very highly conserved (>99% identity) [32], the bread wheat sequence was selected as the reference genome. Thus the reads obtained from all 13 libraries (Additional file 2: Dataset S1) were aligned against the IWGSC draft sequence using the same set of TopHat parameters as given above. The resulting alignments were filtered using the method described by Pfeifer et al. [33].

### Quantification of transcript abundance and the recognition of differential transcription

Transcript abundances were obtained from a set of 99,386 high confidence genes (HC1-HC4) represented in the CS gene model (urgi.versailles.inra.fr/download/iwgsc/Gene_models/). Read numbers were normalized by expressing as read fragments per kilobase of exon model per million mapped base pairs (FPKM) [35], using HTSeq-count v0.6.0 software [34], with the parameters -s and no. Correlations between pairs of samples were based on Pearson’s correlation coefficient, calculated from the “cor” function implemented in the R based on log2 (FPKM + 1) transformed data [33]. A correlation dendrogram of genotypes was generated using the function flashClust. Genes for which the transcript abundance was below unity in both parents and their derived hybrids were considered to be transcribed at too low a level and were removed from the dataset. To identify differentially transcribed genes, the R packages DESeq (for comparisons between samples represented by two replicates) [29, 45] or Ebseq (for comparisons involving LDN) [46] were applied. Genes for which the FDR-adjusted p value was <0.05 were considered to be transcribed at a statistically significant different level.

### Triplet, duplet and singleton genes

The 99,386 high-confidence CS gene models (HC1-HC4) were compared against each other with BLASTP (E-value threshold of 1e^−5^) by considering only alignments with a minimum of 90% sequence similarity as homoeologous genes among the A-, B- and D-subgenome [33, 47]. This produced 28,828 A genome, 30,707 B genome and 29,780 D genome transcripts, which resolved into 8,339 triplets (8,339 × 3 = 25,017 genes/homoeologs), 8,338 duplets (2,370 AB, 3,410 AD, and 2,558 BD) and 47,622 singletons (14,709 A, 17,440 B and 15,473 D) (Additional file 9: Dataset S8). Duplet and triplet genes were considered when searching for genes showing non-additive transcription or ELD, while only the triplet genes were considered in the context of the differential transcription of homoeologues. To compare the transcript abundances between pairs of homoeologues (A/B, A/D, and B/D), read numbers were normalized by dividing by the length of the corresponding gene model before calculating FPKM. To analyze the behaviour of singletons in the allotriploid and allohexaploid plants, a set of 13,505 HC1 genes was considered (of these, 3,992 originated from A genome, 5,614 from B genome and 3,899 from D genome loci, see Additional file 9: Dataset S8).

### Gene Ontology (GO) and MapMan enrichment

The genes were annotated using the best-matched rice gene models (RICE MSU 7.0) [48]. AgriGO (bioinfo.cau.edu.cn/agriGO/) was used for GO analysis [49]. MapMan (mapman.gabipd.org/) was used to identify biological processes and pathways of individual genes and homoeologue sets [50]. GO terms showing a corrected FDR of below 0.05 were considered as being significantly enriched.

## Additional files

Additional file 1: Figure S1. The morphology and cytology of *T. turgidum* LDN (AABB), *A. tauschii* AS60 (DD), the allotriploid LDN × AS60 (ABD) and the derived allohexaploid (AABBDD). **Figure S2.** Global characterization of gene expression patterns among the F1 hybrids and S1 progeny and their progenitors for two compositions. **Figure S3.** Variation in the transcription of homoelogues as a result of allotriploidization and WGD in the LDN × AS60 lineage. **Figure S4.** Non-additive transcription of genes in the allotriploid and allohexaploid in the lineage LDN × AS60. **Figure S5.** The transcription of singletons in the LDN × AS60 lineage. **Table S1.** Statistics for HC1-HC4 gene expression levels. **Table S2.** Classification of 89,315 high confidence Chinese Spring gene models. **Table S3.** Dynamic homoeolog expression patterns for parental expression level dominance genes. **Table S4.** Homoeolog dynamics in F1 and S1 relative to those in their parents. **Table S5.** Comparison of the mapping efficiency of clean reads from *Ae. tauschii* AS60 against different reference genomes. (DOC 571 kb)
Additional file 2:
**Dataset S1.** RNA-seq data statistics and genome mapping rates using Chinese Spring draft genome sequences as references. (XLSX 16 kb)
Additional file 3:
**Dataset S2.** List of differentially expressed homoeologs between F1 and parental lines in LDN × AS60. (XLSX 978 kb)
Additional file 4:
**Dataset S3.** List of differentially expressed homoeologs between S1 and F1 in LDN × AS60. (XLSX 83 kb)
Additional file 5:
**Dataset S4.** List of nonadditively expressed genes in F1 hybrids derived from LDN × AS60. (XLSX 83 kb)
Additional file 6:
**Dataset S5.** List of differentially expressed homoeologs between F1 and parental lines in AS2255 × AS60. (XLSX 793 kb)
Additional file 7:
**Dataset S6.** List of differentially expressed homoeologs between S1 and F1 in AS2255 × AS60. (XLSX 45 kb)
Additional file 8:
**Dataset S7.** List of nonadditively expressed genes in F1 hybrids derived from AS2255 × AS60. (XLSX 72 kb)
Additional file 9:
**Dataset S8.** List of triplet, duplet and singleton genes. (XLSX 3673 kb)

## Abbreviations

AGO4: Argonaute 4
DMS3: Defective in meristem silencing 3
ELD: Expression level dominance
FPKM: Fragments per kilobase per million
GO: Gene ontology
HC: High-confidence
IDN2: INVOLVED IN DE NOVO2
LDN: Langdon
RdDM: RNA-dependent DNA methylation
UTHs: Unequally transcribed homoeologs
WGD: Whole genome doubling
