# Supplementary material for: The abundance of homoeologue transcripts is disrupted by hybridization and is partially restored by genome doubling in synthetic hexaploid wheat
Source: BMC Genomics. 2017 Feb 10;18:149. doi: 10.1186/s12864-017-3558-0 (PMC5303294; doi:10.1186/s12864-017-3558-0)
Supplement: Additional file 1: Figure S1. — The morphology and cytology of T. turgidum LDN (AABB), A. tauschii AS60 (DD), the allotriploid LDN × AS60 (ABD) and the derived allohexaploid (AABBDD). Figure S2. Global characterization of gene expression patterns among the F1 hybrids and S1 progeny and their progenitors for two compositions. Figure S3. Variation in the transcription of homoelogues as a result of allotriploidization and WGD in the LDN × AS60 lineage. Figure S4. Non-additive transcription of genes in the allotriploid and allohexaploid in the lineage LDN × AS60. Figure S5. The transcription of singletons in the LDN × AS60 lineage. Table S1. Statistics for HC1-HC4 gene expression levels. Table S2. Classification of 89,315 high confidence Chinese Spring gene models. Table S3. Dynamic homoeolog expression patterns for parental expression level dominance genes. Table S4. Homoeolog dynamics in F1 and S1 relative to those in their parents. Table S5. Comparison of the mapping efficiency of clean reads from Ae. tauschii AS60 against different reference genomes. (DOC 571 kb) [file 12864_2017_3558_MOESM1_ESM.doc]

**Additional file 1**

**Fig. S1** The morphology and cytology of *T. turgidum* LDN (AABB), *A. tauschii* AS60 (DD), the allotriploid LDN × AS60 (ABD) and the derived allohexaploid (AABBDD). (a) Fluorescent *in situ* hybridization (FISH) analysis of the 21 univalents presents at meiosis metaphase I in the meiocyte of an allotriploid plant. The probe 6C6-3 hybridizing to the centromeres fluoresced green. Bar: 10 µm. (b) Allotriploid pollen mother cells comprise dyads. (c) Multi-colour genomic *in situ* hybridization of a root tip mitotic cell from an allohexaploid plant, showing 2*n=*6*x*=42. (d) Sequential multi-colour FISH of a root tip mitotic cell from an allohexaploid plant, showing that chromosomes of the A, B and D genome were all represented on basis of probes pSc119.2 (green), pAs1 (red), and pTa71(yellow). (e) Morphology of 120 day old plants of AS2255, AS60 and their derived allotriploid (F1) and allohexaploid (S1-S4). (f) Leaf width and length of the first four leaves of the plants. Whiskers indicate SD (*n=*12).

**
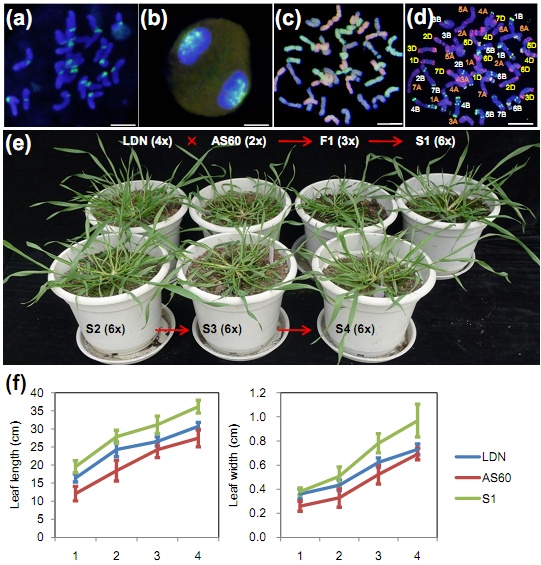
**

**Fig. S2** Global characterization of gene expression patterns among the F1 hybrids and S1 progeny and their progenitors for two compositions. AS2255 and LDN, *T. turgidum*; AS60, *Ae. tauschii*; F1, the triploid hybrid; S1, the first generation of self-pollinated allohexaploid wheat. (**a**) Correlation coefficients between gene expression datasets from two biological duplicates. (**b**-**c**) Cluster dendrogram showing global relationships of gene expression in different genotypes. The branch length indicates the degree of variance. (**d-e**) Gene expression patterns specific for A, B, and D genomes in two composition F1 hybrids and S1 progeny and their progenitors. (**f**) Expressed gene numbers specific for the A, B, and D genomes in the F1 hybrids and S1 progeny and their progenitors for two compositions.

**
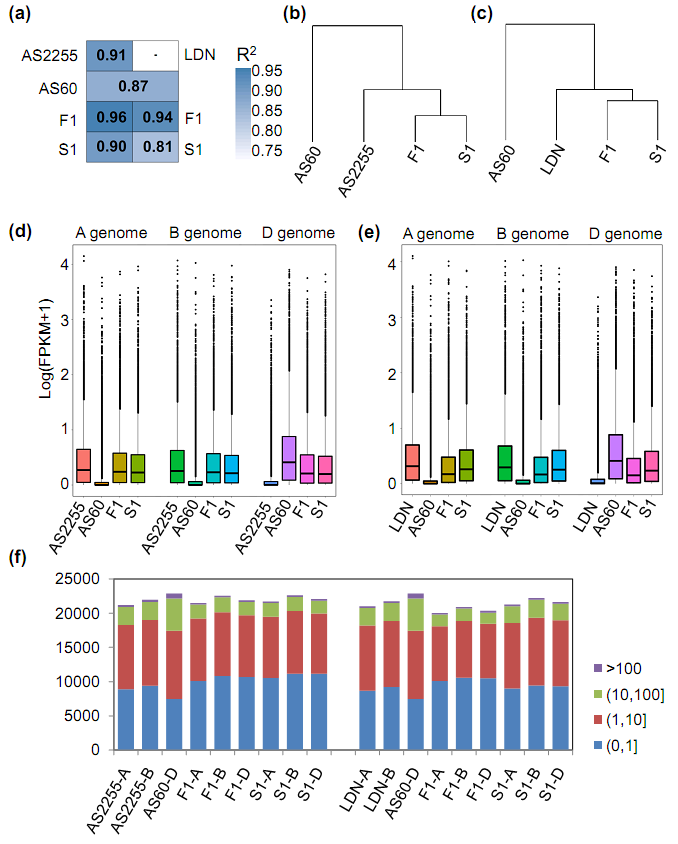
**

**Fig. S3** Variation in the transcription of homoelogues as a result of allotriploidization and WGD in the LDN × AS60 lineage. (a) Differentially transcribed homoeologues. The number next to the symbol for the species represents the number of differentially up-regulated homoeologues vs. the neighboring species linked by a line. A consistent colour has been used to refer to each genome (A genome: blue, B genome: yellow, D genome: purple). Numbers in the middle of each line represent the total numbers of differentially transcribed homoeologues (black). (b) Boxplots illustrating the effect of allotriploidization and WGD on transcript abundance: homoeologues from (1) the A genome, (2) the B genome, (3) the D genome. Differentially transcribed D genome homoeologues between the allotriploid and parent that were transmitted into allohexaploid are used as controls (4). Boxes span the data range between the first and third quartiles, and the median is represented as a horizontal line. Whiskers extend to the most extreme data point, which is no more than 1.5 times the interquartile range away from the first and third quartiles. The widths of the boxes are proportional to the gene numbers.

**
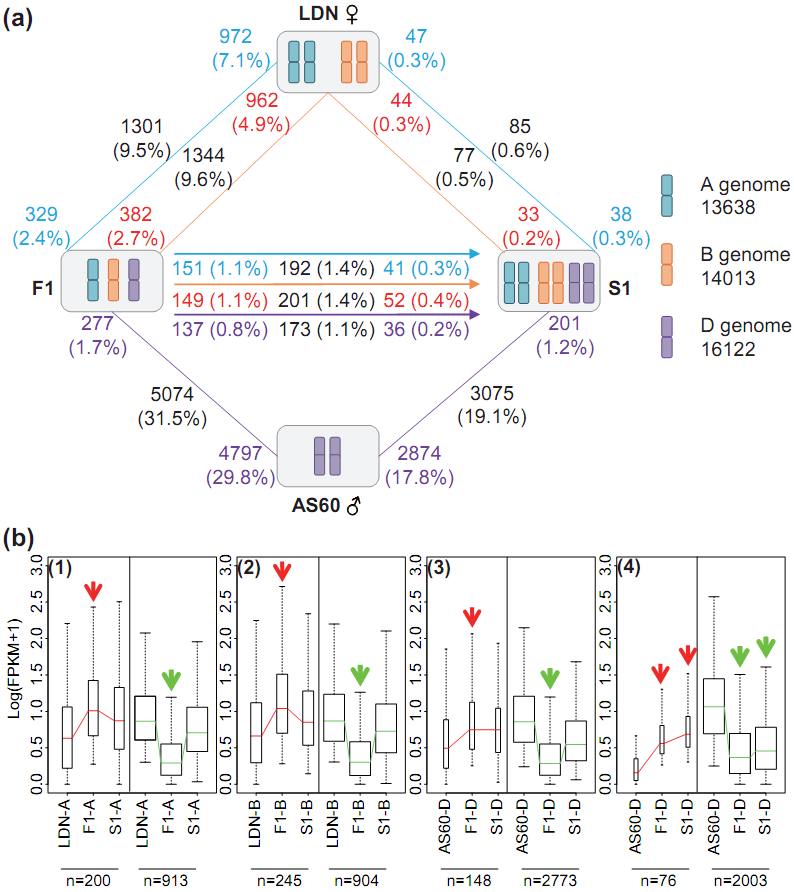
**

**Fig. S4** Non-additive transcription of genes in the allotriploid and allohexaploid in the lineage LDN × AS60. (a) Numbers of non-additively transcribed genes in the progeny compared to mid-parent value (MPV). The red numbers shown refer to genes up-regulated (bottom) or down-regulated (top) in the allotriploid (F1) and allohexaploid (S1). (b) The number of non-additive genes common to the allotriploid and allohexaploid. GO enrichment terms for the genes non-additively genes in the allotriploid are shown below the figure. (c) Homoeologue expression patterns of non-additively expressed genes. “Up” and “down” refer to homoeologues differentially transcribed between the progeny and the parents, whereas “no change” implies that the transcription levels were statistically unchanged by either the allotriploidization or the WGD.

**
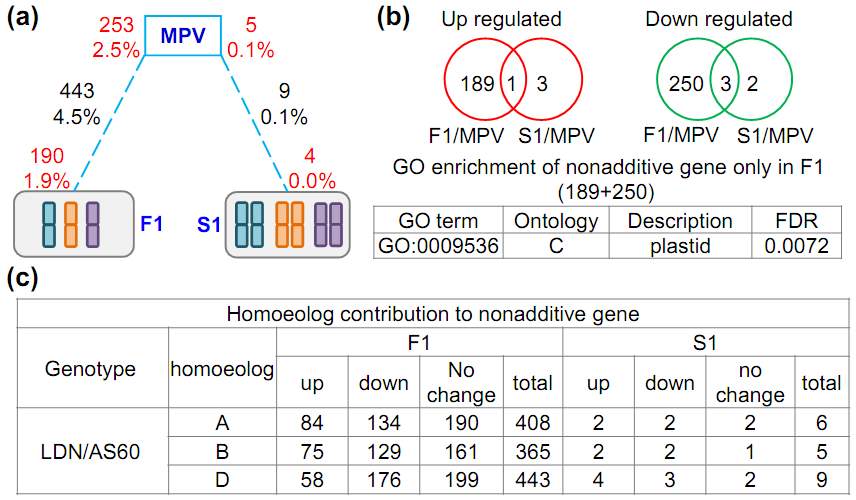
**

**Fig. S5** The transcription of singletons in the LDN × AS60 lineage. (a) Singletons classified according to genome origin; enriched GO terms found in the shared singleton genes are shown below the Venn diagram. (b) The function of singletons derived from the MapMan program.

**
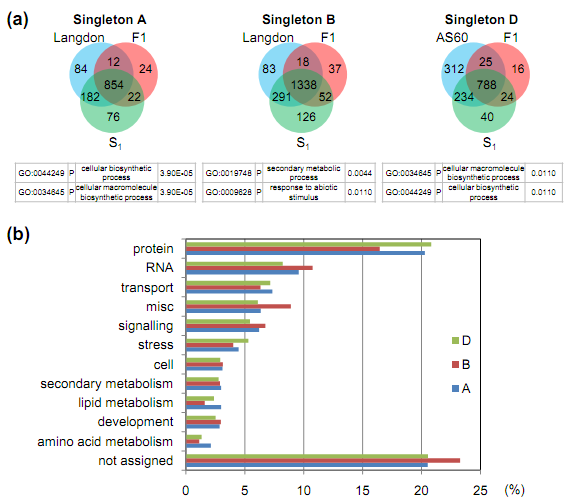
**

**Table S1** Statistics for HC1-HC4 gene expression levels.

| Genotypes | Genes with FPKM>0 | Genes expression level | Expressed genes (FPKM>1) |
| --- | --- | --- | --- |
|  |  | mean |  |
| AS60 (DD) | 22877 | 20.97 ± 175.79 | 15418 |
| AS2255 (AABB) | 43149 | 11.57 ± 156.24 | 24893 |
| AS2255×AS60 F1 (ABD) | 65892 | 8.02 ± 84.87 | 34307 |
| AS2255×AS60 S1 (AABBDD) | 66372 | 8.25 ± 104.79 | 33559 |
| LDN (AABB) | 42739 | 11.47 ± 124.73 | 27531 |
| LDN×AS60 F1 (ABD) | 61282 | 9.42 ± 125.37 | 30137 |
| LDN×AS60 S1(AABBDD) | 65085 | 8.29 ± 87.23 | 37321 |

**Table S2** Classification of 89,315 high confidence Chinese Spring gene models.

| Materails | Singleton genes | | | | Duplet genes | | | | Triplet genes | Unclassified genes | | | |
| --- | --- | --- | --- | --- | --- | --- | --- | --- | --- | --- | --- | --- | --- |
|  | A | B | D | total | AB | AD | BD | total |  | A | B | D | total |
| Chinese Spring | 14709 | 17440 | 15473 | 47622 | 4740 | 6820 | 5116 | 16676 | 25017 |  |  |  |  |
| AS2255/AS60 combination | 4696 (31.9%) | 5355 (30.7%) | 5938 (38.4%) | 15989 (33.6%) | 2113 (44.6%) | 3359 (49.3%) | 2457 (48.0%) | 7929 (47.5%) | 15568 (62.2%) | 1273 | 1454 | 1424 | 4151 |
| LDN/AS60 combination | 4926 (33.5%) | 5635 (32.3%) | 6082 (39.3%) | 16643 (34.9%) | 2198 (46.4%) | 3459 (50.7%) | 2543 (49.7%) | 8200 (49.2%) | 16041 (64.1%) | 1270 | 1468 | 1403 | 4141 |

**Table S3** Dynamic homoeolog expression patterns for parental expression level dominance genes.

| AS2255/AS60 | | ELD-d | | | | | | | | ELD-ab | | | | | | | |
| --- | --- | --- | --- | --- | --- | --- | --- | --- | --- | --- | --- | --- | --- | --- | --- | --- | --- |
|  |  | Ⅱ | | | | Ⅺ | | | | Ⅳ | | | | Ⅸ | | | |
|  |  | AB-HY-D | | | | AB-HY-D | | | | AB-HY-D | | | | AB-HY-D | | | |
|  |  | 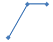   \|  \| \| --- \| | | | | 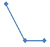   \|  \| \| --- \| | | | | 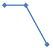   \|  \| \| --- \| | | | | 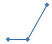   \|  \| \| --- \| | | | |
|  |  | up | down | no change | total | up | down | no change | total | up | down | no change | total | up | down | no change | total |
| F1 | A | 4 | 0 | 162 | 166 | 0 | 14 | 5 | 19 | 3 | 5 | 846 | 854 | 0 | 5 | 395 | 400 |
|  | B | 8 | 0 | 131 | 139 | 0 | 12 | 6 | 18 | 8 | 2 | 781 | 791 | 1 | 6 | 371 | 378 |
|  | D | 5 | 43 | 198 | 246 | 0 | 6 | 20 | 26 | **83** | **23** | **878** | 984 | **0** | **426** | **25** | 451 |
| S1 | A | 3 | 0 | 135 | 138 | 0 | 6 | 4 | 10 | 3 | 0 | 741 | 744 | 1 | 1 | 342 | 344 |
|  | B | 6 | 0 | 113 | 119 | 0 | 4 | 3 | 7 | 5 | 0 | 697 | 702 | 0 | 1 | 305 | 306 |
|  | D | 1 | 30 | 177 | 208 | 0 | 4 | 8 | 12 | **49** | **13** | **808** | 870 | **0** | **331** | **44** | 375 |
| LDN/AS60 | | up | down | no change | total | up | down | no change | total | up | down | no change | total | up | down | no change | total |
| F1 | A | 21 | 0 | 138 | 159 | 0 | 82 | 45 | 127 | 6 | 10 | 536 | 552 | 1 | 17 | 234 | 252 |
|  | B | 26 | 0 | 109 | 135 | 0 | 60 | 38 | 98 | 5 | 9 | 516 | 530 | 0 | 6 | 214 | 220 |
|  | D | 4 | 42 | 168 | 214 | 0 | 39 | 110 | 149 | **46** | **14** | **597** | 657 | **0** | **259** | **24** | 283 |
| S1 | A | 1 | 0 | 34 | 35 | 0 | 1 | 3 | 4 | 0 | 0 | 480 | 480 | 0 | 0 | 193 | 193 |
|  | B | 2 | 0 | 24 | 26 | 0 | 3 | 1 | 4 | 0 | 0 | 460 | 460 | 0 | 0 | 166 | 166 |
|  | D | 0 | 6 | 49 | 55 | 0 | 1 | 7 | 8 | **40** | **6** | **530** | 576 | **0** | **187** | **26** | 213 |

**Table S4** Homoeolog dynamics in F1 and S1 relative to those in their parents.

| **AS2255/AS60** | Singleton A | | | | Singleton B | | | | Singleton D | | | |
| --- | --- | --- | --- | --- | --- | --- | --- | --- | --- | --- | --- | --- |
|  | F1>P | F1<P | F1=P | total | F1>P | F1<P | F1=P | total | F1>P | F1<P | F1=P | total |
|  | 76 | 134 | 4351 | 4561 | 143 | 120 | 4945 | 5208 | 154 | **1781** | 3932 | 5867 |
| S1>P | 29 | 0 | 20 | 49 | 39 | 1 | 21 | 61 | 82 | 0 | 19 | 101 |
| S1<P | 0 | 8 | 8 | 16 | 0 | 5 | 18 | 23 | 0 | **1115** | 198 | 1313 |
| S1=P | 47 | 126 | 4323 | 4496 | 104 | 114 | 4906 | 5124 | 72 | 666 | 3715 | 4453 |
| **LDN/AS60** | Singleton A | | | | Singleton B | | | | Singleton D | | | |
|  | F1>P | F1<P | F1=P | total | F1>P | F1<P | F1=P | total | F1>P | F1<P | F1=P | total |
|  | 149 | 338 | 4114 | 4601 | 182 | 404 | 4629 | 5215 | 125 | 1581 | 4148 | 5854 |
| S1>P | 10 | 0 | 8 | 18 | 14 | 0 | 6 | 20 | 45 | 0 | 48 | 93 |
| S1<P | 0 | 20 | 4 | 24 | 0 | 22 | 7 | 29 | 2 | 762 | 362 | 1126 |
| S1=P | 139 | 318 | 4102 | 4559 | 168 | 382 | 4616 | 5166 | 78 | 819 | 3738 | 4635 |
|  | | | | | | | | | | | | |
| **AS2255/AS60** | Duplet A | | | | Duplet B | | | | Duplet D | | | |
|  | F1>P | F1<P | F1=P | total | F1>P | F1<P | F1=P | total | F1>P | F1<P | F1=P | total |
|  | 41 | 43 | 2463 | 2547 | 47 | 43 | 2048 | 2138 | 63 | 1090 | 1995 | 3148 |
| S1>P | 7 | 0 | 7 | 14 | 13 | 0 | 8 | 21 | 28 | 0 | 8 | 36 |
| S1<P | 0 | 4 | 10 | 14 | 0 | 6 | 7 | 13 | 0 | 716 | 165 | 881 |
| S1=P | 34 | 39 | 2446 | 2519 | 34 | 37 | 2033 | 2104 | 35 | 374 | 1822 | 2231 |
| **LDN/AS60** | Duplet A | | | | Duplet B | | | | Duplet D | | | |
|  | F1>P | F1<P | F1=P | total | F1>P | F1<P | F1=P | total | F1>P | F1<P | F1=P | total |
|  | 80 | 224 | 2247 | 2551 | 85 | 168 | 1882 | 2135 | 72 | 1051 | 2039 | 3162 |
| S1>P | 5 | 0 | 2 | 7 | 4 | 0 | 2 | 6 | 22 | 0 | 14 | 36 |
| S1<P | 0 | 10 | 1 | 11 | 0 | 5 | 4 | 9 | 1 | 453 | 180 | 634 |
| S1=P | 75 | 214 | 2244 | 2533 | 81 | 163 | 1876 | 2120 | 49 | 598 | 1845 | 2492 |
|  | | | | | | | | | | | | |
| **AS2255/AS60** | Triplet A | | | | Triplet B | | | | Triplet D | | | |
|  | F1>P | F1<P | F1=P | total | F1>P | F1<P | F1=P | total | F1>P | F1<P | F1=P | total |
|  | 16 | 59 | 4870 | 4944 | 23 | 52 | 4783 | 4858 | 72 | 1935 | 3659 | 5666 |
| S1>P | 2 | 0 | 7 | 9 | 4 | 0 | 3 | 7 | 30 | 0 | 5 | 35 |
| S1<P | 0 | 6 | 10 | 16 | 0 | 5 | 4 | 9 | 0 | 1115 | 203 | 1318 |
| S1=P | 14 | 53 | 4853 | 4920 | 19 | 47 | 4776 | 4842 | 42 | 820 | 3451 | 4313 |
| **LDN/AS60** | Triplet A | | | | Triplet B | | | | Triplet D | | | |
|  | F1>P | F1<P | F1=P | total | F1>P | F1<P | F1=P | total | F1>P | F1<P | F1=P | total |
|  | 88 | 387 | 4521 | 4996 | 93 | 367 | 4465 | 4925 | 69 | 1878 | 3694 | 5641 |
| S1>P | 1 | 0 | 1 | 2 | 2 | 0 | 0 | 2 | 11 | 1 | 9 | 21 |
| S1<P | 0 | 6 | 3 | 9 | 0 | 3 | 3 | 6 | 0 | 637 | 261 | 898 |
| S1=P | 87 | 381 | 4517 | 4985 | 91 | 364 | 4462 | 4917 | 58 | 1240 | 3424 | 4722 |
| Significance by Benjamini-Hchberg multiple test correction (P < 0.05) | | | | | | | | | | | | |

**Table S5** Comparison of the mapping efficiency of clean reads from *Ae. tauschii* AS60 against different reference genomes.

| Reference genome | Map to genome | Repeat 1 | | Repeat 2 | |
| --- | --- | --- | --- | --- | --- |
|  |  | reads number | percentage | reads number | percentage |
| *Aegilops tauschii* (DD ) | clean reads | 49,311,018 |  | 54,996,602 |  |
|  | mapped reads | 37,137,487 | 75.31% | 43,903,405 | 79.83% |
| Chinese Spring (AABBDD) draft genome  sequences (IWGSC) | mapped reads | 35,334,697 | 71.66% | 42,170,505 | 76.68% |
|  | reads mapped to A subgenome | 2,336,601 | 4.74% | 3,638,024 | 6.61% |
|  | reads mapped to B subgenome | 3,098,353 | 6.28% | 4,435,609 | 8.07% |
|  | reads mapped to D subgenome | 20,438,631 | 41.45% | 25,209,893 | 45.84% |
|  | reads mapped to AB subgenome | 1,166,919 | 2.37% | 1,465,445 | 2.66% |
|  | reads mapped to AD subgenome | 3,023,023 | 6.13% | 2,642,727 | 4.81% |
|  | reads mapped to BD subgenome | 2,875,044 | 5.83% | 2,367,536 | 4.30% |
|  | reads mapped to ABD subgenome | 2,396,126 | 4.86% | 2,411,271 | 4.38% |
